# Molecular characterization of Kita-Kyushu lung cancer antigen (KK-LC-1) expressing carcinomas

**DOI:** 10.18632/oncotarget.28132

**Published:** 2021-12-07

**Authors:** Robert Hsu, Yasmine Baca, Joanne Xiu, Rongfu Wang, J. Nicholas Bodor, Chul Kim, Hina Khan, Hirva Mamdani, Misako Nagasaka, Sonam Puri, Stephen V. Liu, W. Michael Korn, Jorge J. Nieva

**Affiliations:** ^1^Department of Internal Medicine, Division of Medical Oncology, Norris Comprehensive Cancer Center and Hospital, University of Southern California, Los Angeles, California, USA; ^2^Caris Life Sciences, Phoenix, Arizona, USA; ^3^Department of Pediatrics, Children’s Hospital Los Angeles, Keck School of Medicine, University of Southern California, Los Angeles, California, USA; ^4^Department of Hematology/Oncology, Fox Chase Center, Philadelphia, Pennsylvania, USA; ^5^Division of Hematology and Oncology, Georgetown Lombardi Comprehensive Cancer Center, Washington, District of Columbia, USA; ^6^Department of Internal Medicine, Division of Hematology and Oncology, The Warren Alpert Medical School of Brown University, Providence, Rhode Island, USA; ^7^Department of Oncology, Wayne State University School of Medicine and The Barbara Karmanos Cancer Institute, Detroit, Michigan, USA; ^8^Division of Neurology, Department of Internal Medicine, St. Marianna University, Kawasaki, Kanagawa, Japan; ^9^Division of Medical Oncology, Huntsman Cancer Institute, University of Utah, Salt Lake City, Utah, USA

**Keywords:** lung cancer, tumor microenvironment, diagnostic biomarkers, biomarkers for immunotherapy, cancer testis antigen

## Abstract

Cancer/testis antigens (CTAs) are strongly expressed in some solid tumors but minimally expressed in normal tissue, making them appealing therapeutic targets. KK-LC-1 (CXorf61) has cytoplasmic expression in gastric, breast, and lung cancer. We characterized the molecular subtypes of non-small cell lung cancer (NSCLC) expressing KK-LC-1 to inform rational clinical trials of T-cell receptor therapy (TCR-T) targeting KK-LC-1. 9790 NSCLC tumors that underwent whole transcriptome sequencing (Illumina NovaSeq) and NextGen DNA sequencing (NextSeq, 592 Genes and NovaSEQ, WES) at Caris Life Sciences (Phoenix, AZ) were analyzed. Tumors were split into quartiles based on KK-LC-1 expression and pathological and molecular differences were investigated. Adenocarcinoma had significantly higher KK-LC-1 expression than squamous cell carcinoma (median, 3.25 vs. 1.17 transcripts per million (TPM), *p* < 0.0001). Tumors with the highest quartile of KK-LC-1 expression had a greater proportion of tumors with high tumor mutation burden (TMB) (≥10 mutations per megabase; 44% vs. 28% in Q1, *p* < 0.001). Increased KK-LC-1 expression was associated with increased M1 macrophage abundance. Higher levels of KK-LC-1 expression were seen in pan-wild type and *KRAS* mutated tumors and associated with high TMB. TCR-T therapy directed against KK-LC-1 should be considered in patients whose clinical features reflect these characteristics.

## INTRODUCTION

Cancer testis antigens (CTA) are germ cell antigens that are typically minimally expressed in normal tissues, but commonly expressed in tumors. Because of this, they are appealing therapeutic targets with less potential for off target toxicity [1]. Kita-Kyushu lung cancer antigen-1 (KK-LC-1), also known as CT 83 and CXORF61, is a CTA containing 113 amino acids that maps to chromosome Xq22 and is not expressed in normal tissues except for the testis. It has been shown to be expressed in 82% of gastric cancer tumors and 52.9% of breast cancer tumors, with even higher detection rates in triple negative breast cancer [2, 3]. In lung cancer, surgical series have shown KK-LC-1 to be expressed in about one-third of lung cancer tumors [1, 3–5]. In one study with Japanese patients, KK-LC-1 has been shown to be expressed at similar rates in adenocarcinoma and squamous cell carcinoma [5]. KK-LC-1 high expression was shown in a greater proportion of TNM stage II and III tumors compared to TNM stage I tumors [1]. There are no large series studying the prevalence of the disease in patients with stage IV NSCLC.

Cytotoxic T lymphocytes (CTLs) are an emerging modality for immunity against cancers, as antigen specific CTLs can be formulated to specifically target CTA expressing tumor cells. T-cell receptor therapy is designed to modify patient T cells with a selected T-cell receptor targeting a cancer testis antigen which can promote selective targeting of tumor antigens while limiting off target toxicity. One challenge is that not all patients have T cells that will effectively recognize their tumors. TCR-Ts in order to be effective need a high affinity towards the cancer testis antigen and many times the T cells that would be designed to recognize these tumors are eliminated during the negative selection process in the thymus [6–8]. Fukuyama et al. identified the KK-LC-1 CTA by establishing a lung adenocarcinoma cell line and induced a CTL clone from regional lymph node lymphocytes of a patient [9]. More recently, Marcinkowski et al. identified a KK-LC-1 reactive T cell receptor from tumor infiltrating lymphocytes (TIL) in a patient with cervical cancer who had a subsequent complete tumor response to TIL therapy [10]. Paret et al. also showed strong antigen-specific response in HLA-A*02 transgenic mice with KK-LC-1 encoding RNA in triple negative breast cancer cell lines. [11]. In addition, other cancer testis antigens such as MAGE-A4 and NY-ESO-1 have shown promise in lung cancer [6, 12].

The majority of non-small cell lung cancer (NSCLC) is diagnosed at an advanced, incurable stage. Five-year relative survival rate for all stages combined is 21% and only 6% for lung cancer that has spread to distant sites, according to SEER data from 2009–2015 [13]. Lung cancer treatment has made tremendous strides in the past decade with advances in targeted therapies for *EGFR, ALK, ROS1, BRAF V600E, MET* exon 14 skip mutation, *RET, NTRK*, and most recently *KRAS* *G12C* along with checkpoint inhibitor immunotherapy in other variants of the disease. Targeted therapies have shown a median progression free survival of around 9–18 months with long term results showing 5-year survival of greater than 14% in *EGFR* patients and 4-year survival in *ALK* and *ROS1* gene rearrangement of 56.6% and 51.1% respectively [14–20]. While immunotherapy has shown significant improvements, standard of care immunotherapy with chemotherapy showed an ORR of 48% and a median PFS of 9 months and OS of 22 months [21].

Because the treatment algorithms have diverged between lung cancer subtypes that are amenable to targeted therapy or immunotherapy, positioning emerging treatments for future clinical trials requires understanding of which molecular subtype of lung cancer would be most appropriate for the new CTA directed immune treatments. Therefore, the aim of this study is to identify the molecular subtypes of lung cancer expressing KK-LC-1 to determine which patients would be most likely to benefit from a clinical trial of T cell receptor therapy (TCR-T) targeting KK-LC-1.

## RESULTS

The study population included a total of 9790 tumors with KK-LC-1 expression ranging from 0–266 TPM. Tumors were then categorized into four groups based on quartiles of KK-LC-1 expression (Supplementary Table 1). We observed a relatively equal median age of patients across all four quartiles of KK-LC-1 expression (Supplementary Table 1). Overall, there seemed to be a slightly higher prevalence of males in the lowest quartile (Q1) of KK-LC-1 expression and a higher prevalence of females in the highest quartile (Q4) of KK-LC-1 expression. (Table 1) We see an even greater proportion of adenocarcinoma tumors (1730/2448, 70.6%) in the tumors in Q4 compared to tumors (1294/2448, 52.9%) in Q1 (Supplementary Table 2). Adenocarcinoma tumors were mostly pan-wild type (36.5%), *KRAS* mutated (35.4%), and *EGFR* mutated (16.3%) while squamous cell carcinoma tumors were even more predominantly pan-wild type (85.5%) (Supplementary Figure 1).

**Table 1:** Baseline characteristics of 9790 NSCLC patients

KK-LC-1 expression quartiles	Total	Female N (%)	Male N (%)	Median age
**Q1**	2448	1138 (46.5)	1310 (53.5)	69
**Q2**	2447	1295 (52.9)	1152 (47.1)	69
**Q3**	2447	1311 (53.6)	1136 (46.4)	69
**Q4**	2448	1320 (53.9)	1128 (46.1)	68
**Total**	**9790**	**5064 (51.7)**	**4726 (48.3)**	**69**

**Figure 1:**
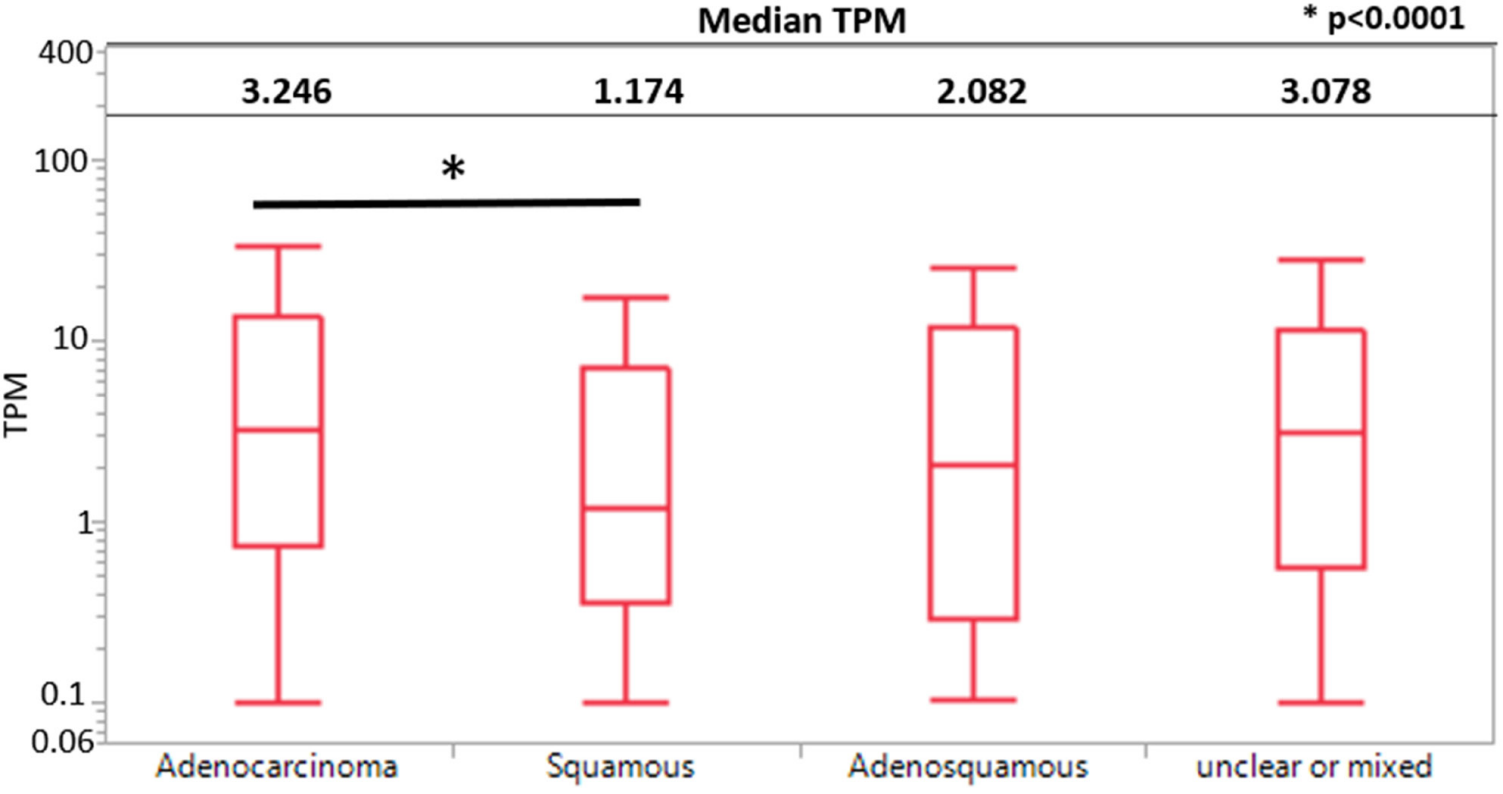
KK-LC-1 expression by histological subtypes between adenocarcinoma, squamous cell carcinoma, adenosquamous carcinoma, and unclear or mixed carcinomas using a boxplot. TPM expression is represented here using a log scale.

Adenocarcinomas had significantly higher median KK-LC-1 expression than squamous cell carcinomas (3.25 vs. 1.17 transcripts per million (TPM), *p* < 0.0001). We also saw a significant difference between squamous cell carcinoma and unclear or mixed (1.14 vs. 3.078 TPM, *p* < 0.001). KK-LC-1 expression between other subtypes were not statistically different (Figure 1).

**Figure 2:**
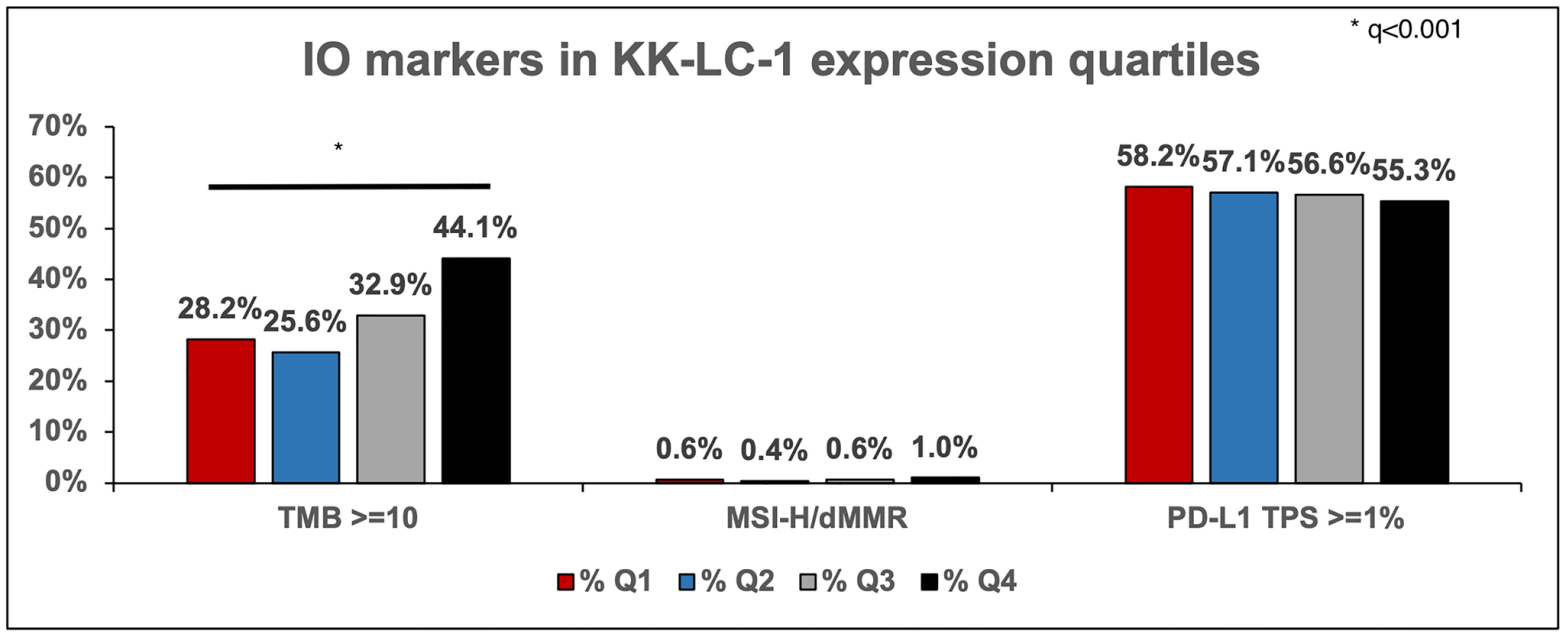
IO markers in tumors in their respective quartile of KK-LC-1 expression that have TMB >= 10, MSI-H/dMMR, and PD-L1 with TPS >= 1%.

Tumors within the highest quartile of KK-LC-1 expression (Q4) had a greater proportion of TMB > 10 mutations per megabase (mt/MB) (44% vs. 28%) compared to Q1. No difference was seen in PD-L1 expression (Figure 2). In adenocarcinoma, Q4 had a higher TMB (9 vs. 5 in Q1).

**Figure 3:**
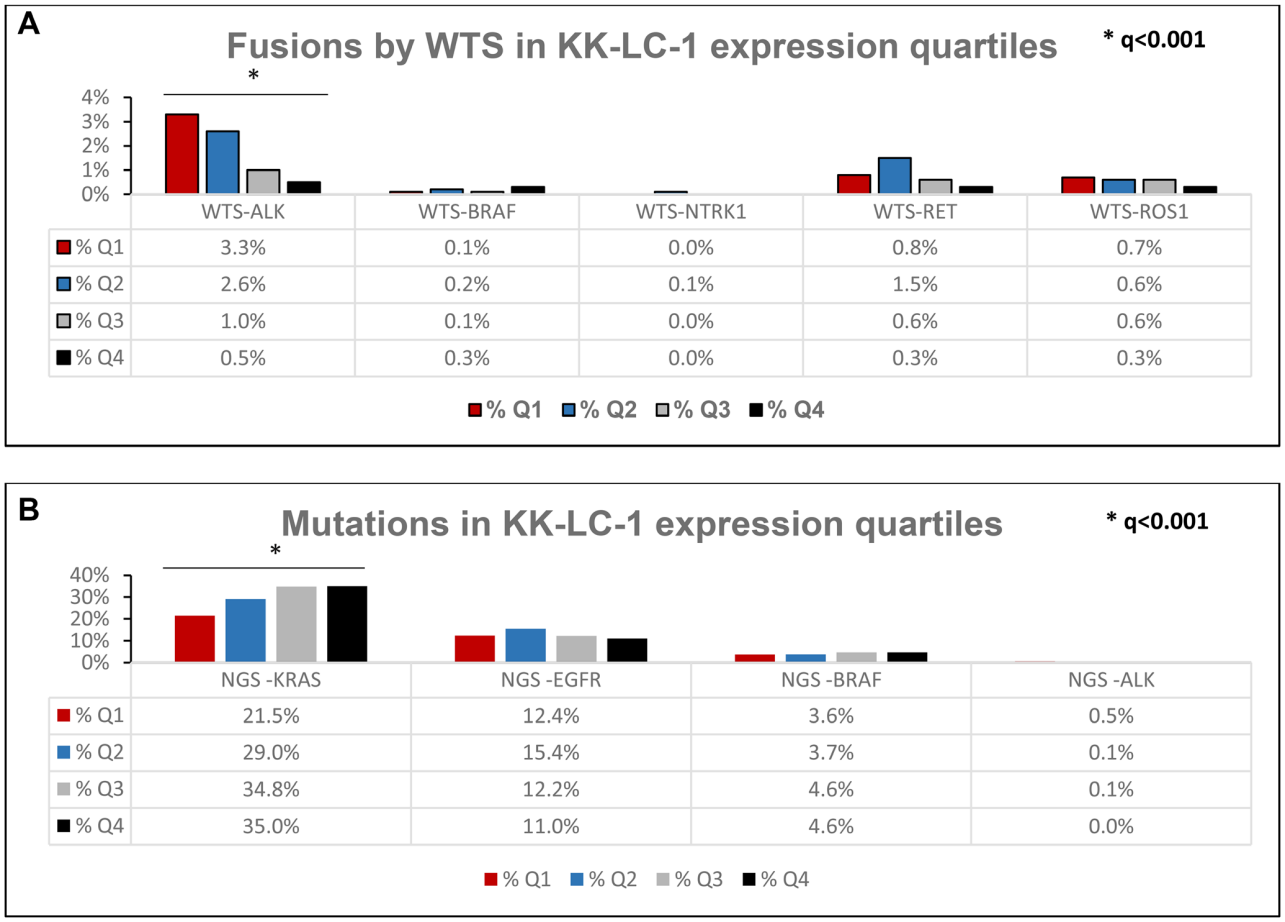
(**A**) Prevalence of fusions based on KK-LC-1 expression quartiles. (**B**) Prevalence of oncogenic mutations based on KK-LC-1 quartiles. Further breakdown of *KRAS*, *EGFR*, and *BRAF* mutations are noted in Supplementary Tables 3–5. NGS-ALK mutations refer to pathogenic/likely *ALK* mutations.

**Figure 4:**
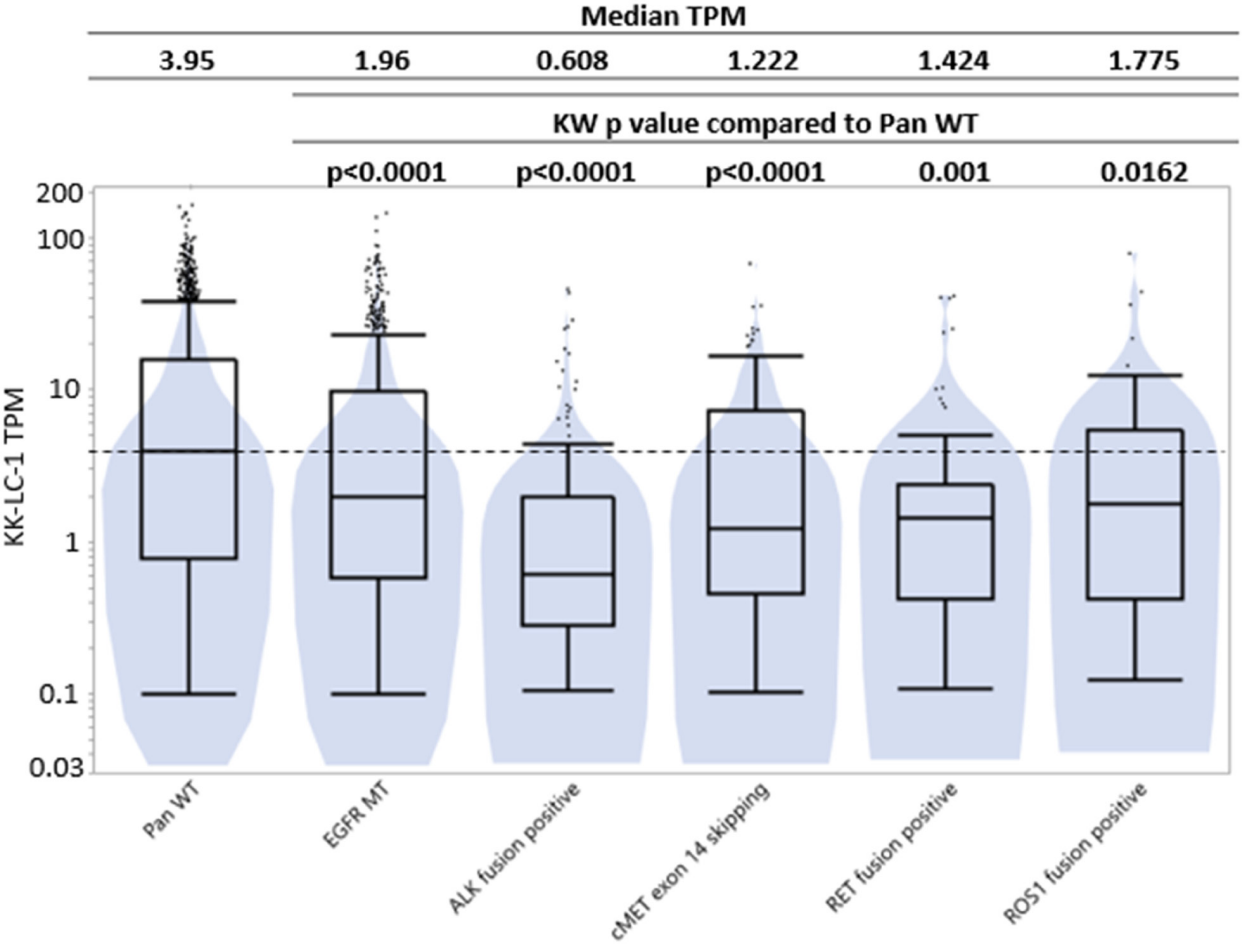
KK-LC-1 expression by transcripts per million (log-scale) based on whether NSCLC tumors were pan-wild type, *EGFR* mutant, *ALK* fusion positive, *cMET* exon 14 skipping, *RET* fusion positive, and *ROS1* fusion positive tumors. TPM expression is represented here using a log scale.

There was a statistically higher *KRAS* mutation prevalence in Q3/Q4 (34.8%/35.0%) than Q1/Q2 (22%/29%) but a lower *ALK* fusion prevalence in Q3/4 (1.0%/0.5%) compared to Q1/2 (3.3%/2.6%) (Figure 3A, 3B). There is statistically higher expression of KK-LC-1 in pan wild type (3.95 TPM) compared to tumors with *EGFR* mutation (1.95 TPM, *p* < 0.0001), *ALK* fusion (0.6 TPM, *p* < 0.0001), *MET* exon-14-skip (1.22 TPM, *p* < 0.0001), *RET* fusion (1.42 TPM, *p* = 0.0001), and *ROS1* fusion (1.78 TPM, *p* = 0.0162) (Figure 4).

Actionable mutations (*ALK, RET, ROS1*) are significantly more prevalent in KK-LC-1 low expressors (Q1) where as *KRAS* mutations are significantly more prevalent in KK-LC-1 high expressors (Q4) (Supplementary Figures 2 and 3).

Specifically, *KRAS* G12C mutations trended higher in KK-LC-1 Q4 when compared to Q1 (*p* = 0.0258) (Supplementary Table 3). There was no significance in number of *EGFR* sensitizing or resistant mutations when comparing KK-LC-1 Q1 vs. Q4. (Supplementary Table 4) In addition, no significance in difference among *BRAF* mutations was seen across the KK-LC-1 expression quartiles (Supplementary Tables 5 and 6).

**Figure 5:**
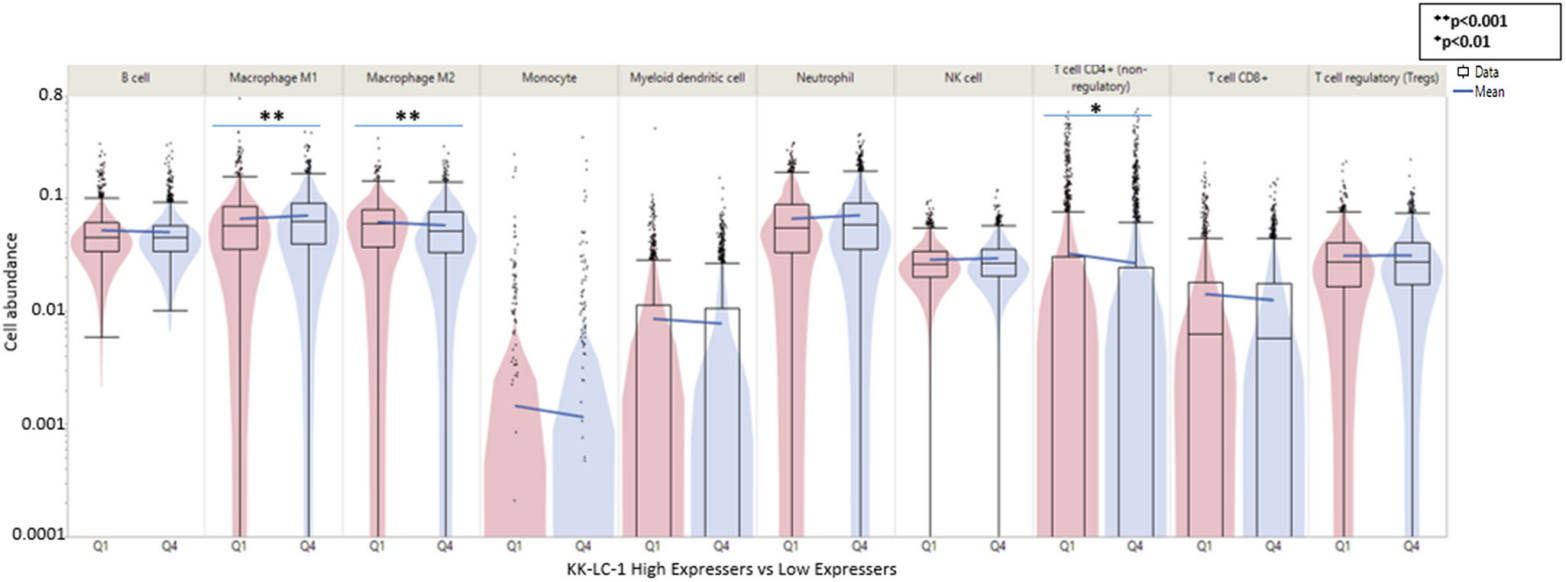
Tumor microenvironment in KK-LC-1 high vs low expressing (Q4 vs. Q1) in adenocarcinomas as measured by immune cell fraction calculated by QuantiSeq. The elements of the tumor microenvironment calculated include B cell, M1 macrophage, M2 macrophage, monocytes, myeloid dendritic cells, neutrophils, NK cells, CD4+ T cells, CD8+ T cells, and regulatory T cells.

When comparing Q4 vs. Q1 in the tumor microenvironment for KK-LC-1, we see a greater proportion of M1 macrophages in Q4 tumors whereas there is a lower proportion of M2 macrophages and CD4+ T cells (Figure 5, Supplementary Figure 4).

## DISCUSSION

Models of cancer tumorigenesis have focused cancer cells arising from molecular mutations in mature somatic cells. However, pluripotent stem cells can give rise to either tissue composed of mature somatic tissue or immature embryonic tissue by ectopically induced transcription factors [22]. Both hematopoietic and solid tumor cancer cells have transcriptional activity that mirrors that of pluripotent and multipotent stem cells [23]. These same transcription factors involved in the reprogramming of pluripotent cells have also been shown to be involved *in vivo* in tissue damage and senescence signaling [24]. Thus, it has been hypothesized that genes involved in early-stage embryogenesis and silenced after might be reactivated as a result of an insult or damage [25]. Targeting CTAs provides an opportunity to help patients based on recognition of this biology.

In determining the optimal patient population for future trials of KK-LC-1 targeted T cells, we see that there is a higher tumor mutation burden in NSCLC expressing high levels of KK-LC-1, as evidenced by higher percentages of tumors with TMB ≥10 in the highest quartile expression of KK-LC-1 and greater median TMB in Q4 versus Q1 (9 vs. 6 in Q1) (Figure 2). Also, a significantly higher percentages of KK-LC-1 expression was observed in pan wild type tumors and those with *KRAS* mutations compared to actionable oncogenic mutation tumors such as *EGFR, BRAF, ALK, NTRK, RET, and ROS1* (Figure 3A, 3B).

Meanwhile, in discordance with previous literature, we find that tumors with adenocarcinoma had significantly higher KK-LC-1 expression levels than squamous cell carcinomas (Supplementary Figure 1) [5]. This may be explained by the propensity for patients with molecular profiling to be of more advanced stage and the use of a more diverse North American patient population than prior surgical series [1, 4, 5, 9].

Tumor associated macrophages (TAMs) are involved in creating an immunosuppressive microenvironment. They affect tumor growth, angiogenesis, immune regulation, metastasis, and chemoresistance. M1 macrophages are part of the classically activated macrophage pathway and are known to secrete proinflammatory cytokines and express markers, notably MHCII, CD68, CD80, iNOS, pSTAT. M2 macrophages meanwhile exert an immunosuppressive phenotype, favoring tissue repair and tumor progression; they secrete anti-inflammatory cytokines such as IL-10, IL-13, and IL-4 along with expressing CD206. TAMs tend to acquire a M2-like phenotype acquired macrophages with macrophage polarization between M1 and M2 macrophages [26]. Previous studies have shown that TAMs in NSCLC have been associated with angiogenesis and lymphangiogenesis with high M2 macrophage ratio and *VEGF* expression [27].

In evaluating our analysis, tumors with higher KK-LC-1 expression seem to favor a microenvironment enriched for cells representing innate immunity. There was significantly fewer CD4+ cells in Q4 tumors compared to Q1 and while not statistically significant fewer CD8+ and myeloid dendritic cells as well in Q4 tumors. Q4 tumors also trended to have more neutrophils and NK cells. In addition, we saw significantly greater representation of M1 macrophages while a significantly lower fraction of M2 macrophages was observed in highly KK-LC-1 expressing tumors. Previous therapies shown to induce M1 macrophage polarization include agents used to treat lung cancer in paclitaxel and docetaxel [28]. Anti PD-L1 treatment has been shown to remodel the macrophage compartment towards a pro-inflammatory phenotype favoring M1 macrophage expression [29]. We do not have treatment history available on our cohort, further studies looking at pretreated and untreated cohorts may be beneficial in delineating between which patients would be benefit from targeted therapy towards KK-LC-1.

Our findings show that the KK-LC-1 antigen is associated with a higher mutation burden. This suggests that patients with the highest KK-LC-1 expression are likely those with lung adenocarcinoma without targetable mutations aside from *KRAS* *G12C*. From our findings, the most likely candidates for a clinical trial using TCR-T therapy directed against KK-LC-1 would be patients who have been treated with checkpoint inhibitors and less likely to have been treated with tyrosine kinase inhibitors.

There are several limitations of our study. First is that the study was performed using de-identified patient samples without the benefit of clinical history. Second, we did not include protein expression measurements which would be important for identification of lung cancer patients most likely to benefit from TCR-T therapy. Finally, although we identified those tumors with higher and lower numbers of transcripts for KK-LC-1, we do not know the threshold of gene expression that would be relevant for targeted T-cells or for any other targeted immunotherapy. Establishing this minimal level will require interventional studies with clinical endpoints. Future directions include investigation of other cancer testis antigens particularly those antigens that are candidates for T-cell based immunotherapy [6, 12].

## MATERIALS AND METHODS

### Tumor samples

The study included NSCLC tumor samples submitted to Caris Life Sciences (Phoenix, AZ) for analysis. This study was conducted in accordance with guidelines of the Declaration of Helsinki, Belmont report, and U.S. Common rule. In keeping with 45 CFR 46.101(b)[4], this study was performed utilizing retrospective, deidentified clinical data. Therefore, this study was considered IRB exempt and patient consent was not required.

### mRNA expression (WTS)

KK-LC-1 expression data was evaluated using RNA sequencing. Full formalin-fixed paraffin-embedded (FFPE) specimens underwent review by a board-certified pathologist to measure percent tumor content and tumor size; a minimum of 20% of tumor content in the area for microdissection was required to enable enrichment and extraction of tumor-specific RNA. Qiagen RNA FFPE tissue extraction kit was used for extraction, and the RNA quality and quantity were determined using the Agilent TapeStation. Biotinylated RNA baits were hybridized to the synthesized and purified cDNA targets and the bait-target complexes were amplified in a post capture PCR reaction. The Illumina NovaSeq 6500 was used to sequence the whole transcriptome from patients to an average of 60M reads. Raw data was demultiplexed by Illumina Dragen BioIT accelerator, trimmed, counted, PCR-duplicates removed and aligned to human reference genome hg19 by STAR aligner. For transcription counting, transcripts per million molecules was generated using the Salmon expression pipeline. Human All Exon V7 bait panel (Agilent Technologies, Santa Clara, CA). Additionally, immune cell fraction was calculated by QuantiSeq [30] using this transcriptomic data.

### Immunohistochemistry (IHC)

Immunohistochemistry (IHC) was performed on FFPE sections of glass slides. Slides were stained using automated staining techniques, per the manufacturer’s instructions, and were optimized and validated per CLIA/CAO and ISO requirements. The primary PD-L1 antibody clone was 22c3 (Dako). Tumor Proportion Score (TPS) was measured, defined as the percentage of viable tumor cells showing partial or complete membrane staining at any intensity. The tumor was considered positive if TPS ≥ 1%.

### Next-generation sequencing (NGS)

NGS was performed on genomic DNA isolated from FFPE tumor samples using the NextSeq platform (Illumina, Inc., San Diego, CA). Matched normal tissue was not sequenced. A custom-designed SureSelect XT assay was used to enrich 592 whole-gene targets (Agilent Technologies, Santa Clara, CA). All variants were detected with >99% confidence based on allele frequency and amplicon coverage, with an average sequencing depth of coverage of >500 and an analytic sensitivity of 5%. Prior to molecular testing, tumor enrichment was achieved by harvesting targeted tissue using manual microdissection techniques. Genetic variants were interpreted by molecular geneticists and categorized as “pathogenic,” “presumed pathogenic,” “pathogenic variant”, “variant of unknown significance,” “presumed benign” or “benign” according to the American College of Medical Genetics and Genomics (ACMG) standards. “Pathogenic”, “resumed pathogenic”, and “pathogenic variants” were counted as mutations whereas “benign”, “presumed benign”, and “variants of unknown significance” were excluded. Pan wild type tumors were defined as tumors that did not contain a “pathogenic,” “presumed pathogenic,” or “pathogenic variant” mutation. Specific information on criteria for *BRAF* and *EGFR* mutations are provided (Supplementary Tables 7 and 8).

### Tumor mutational burden (TMB)

TMB was measured by counting all non-synonymous missense, nonsense, inframe insertion/deletion and frameshift mutations found per tumor that had not been previously described as germline alterations in dbSNP151, Genome Aggregation Database (gnomAD) databases or benign variants identified by Caris geneticists. A cutoff point of ≥10 mutations per MB was used to define high TMB. [31] Caris Life Sciences is a participant in the Friends of Cancer Research TMB Harmonization Project [32].

### Microsatellite instability (MSI)

A combination of multiple test platforms was used to determine dMMR/MSI-H status of the tumors profiled, including fragment analysis (FA, Promega, Madison, WI), IHC (MLH1, M1 antibody; MSH2, G2191129 antibody; MSH6, 44 anti-body; and PMS2, EPR3947 antibody [Ventana Medical Systems, Inc., Tucson, AZ, USA]) and NGS (for tumors tested with NextSeq platform, 7,000 target microsatellite loci were examined and compared to the reference genome hg19 from the University of California).

### Data and statistical analysis

Prevalence of molecular alterations among KK-LC-1 expression quartiles were analyzed using Chi-square or Fisher Exact tests. KK-LC-1 TPM distribution among molecular subtypes and histological subtypes were analyzed using non-parametric Kruskal-Wallis testing. Similarly, tumor microenvironment cell fractions were analyzed among expression quartiles as described previously. A value of <0.05 was considered a trending difference; *p* values were further corrected for multiple comparison using Benjamini-Hochberg method to avoid type I error and an adjusted *p* value (i.e., *q* value) of <0.05 was considered a significant difference.

## SUPPLEMENTARY MATERIALS



## Abbreviations

CTAs: Cancer/Testis Antigens
KK-LC-1/CXorf61: Kita-Kyushu Lung Cancer Antigen-1
CT 83: Cancer/Testis Antigen 83
NSCLC: Non-Small Cell Lung Cancer
TCR-T: T-Cell Receptor Therapy
TPM: Transcripts Per Million
EGFR: Epidermal Growth Factor Receptor
ALK: Anaplastic Lymphoma Kinase
KRAS: Kirsten Rat Sarcoma Virus
CTLs: Cytotoxic T Lymphocytes
TIL: Tumor Infiltrating Lymphocytes
MAGE-A4: Melanoma-Associated Antigen A4
NY-ESO-1: New York Esophageal Squamous Cell Carcinoma 1
ROS1: Proto-oncogene tyrosine-protein kinase ROS
BRAF: V-raf murine sarcoma viral oncogene homolog B1
MET: Exon 14 Mesenchymal-to-Epithelial Transition Exon 14
RET: Rearranged During Transfection Proto-oncogene
NTRK: Neurotrophic Tyrosine Receptor Kinase
FFPE: Formalin-Fixed Paraffin-Embedded
IHC: Immunohistochemistry
PD-L1: Programmed Death-Ligand 1
PD-1: Programmed Death-1
TPS: Tumor Proportion Score
NGS: Next-Generation Sequencing
TMB: Tumor Mutational Burden
MSI: Microsatellite Instability
TAMs: Tumor Associated Macrophages
MHC-II: Major Histocompatibility Complex Class II
CD68: Cluster of Differentiation 68
CD80: Cluster of Differentiation 80
iNOS: Inducible Nitric Oxide Synthase
pSTAT: Phosphorylated Signal Transducer and Activator of Transcription
IL-10: Interleukin 10
IL-13: Interleukin 13
IL-4: Interleukin 4
VEGF: Vascular Endothelial Growth Factor
CD4: Cluster of Differentiation 4
CD8: Cluster of Differentiation 8
