# Supplementary material for: Molecular characterization of Kita-Kyushu lung cancer antigen (KK-LC-1) expressing carcinomas
Source: Oncotarget. 2021 Dec 7;12(25):2449–58. doi: 10.18632/oncotarget.28132 (PMC8664394; doi:10.18632/oncotarget.28132)
Supplement: Supplementary file 1 [file oncotarget-12-2449-s001.pdf]

Molecular characterization of Kita-Kyushu lung cancer antigen (KK-LC-1) expressing carcinomas

SUPPLEMENTARY MATERIALS

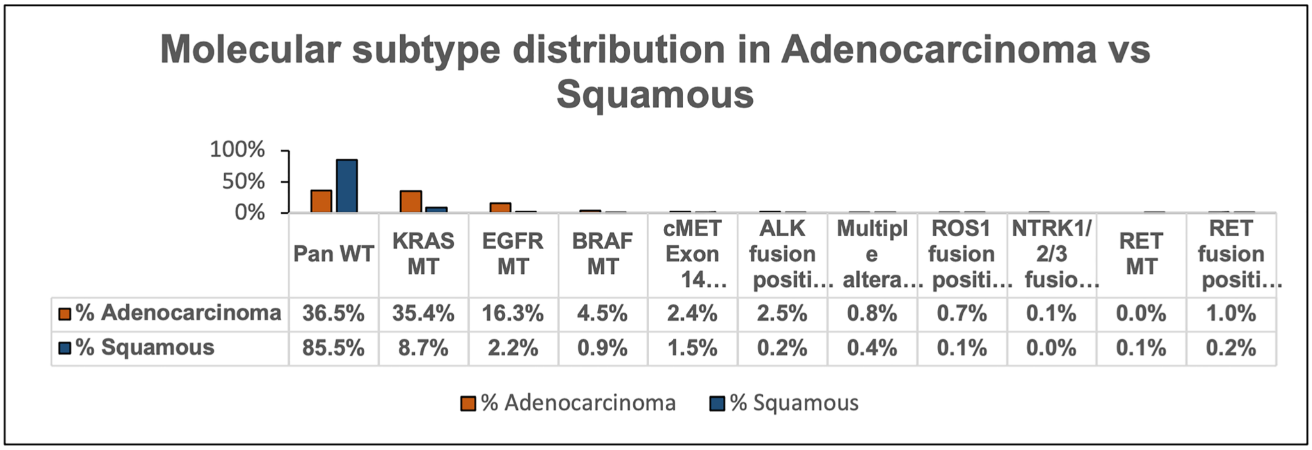

	Pan WT	KRAS MT	EGFR MT	BRAF MT	cMET Exon 14…	ALK fusion positi…	Multipl e altera…	ROS1 fusion positi…	NTRK1/ 2/3 fusio…	RET MT	RET fusion positi…
% Adenocarcinoma	36.5%	35.4%	16.3%	4.5%	2.4%	2.5%	0.8%	0.7%	0.1%	0.0%	1.0%
% Squamous	85.5%	8.7%	2.2%	0.9%	1.5%	0.2%	0.4%	0.1%	0.0%	0.1%	0.2%

Supplementary Figure 1: Molecular subtype distribution between adenocarcinoma and squamous cell carcinoma.

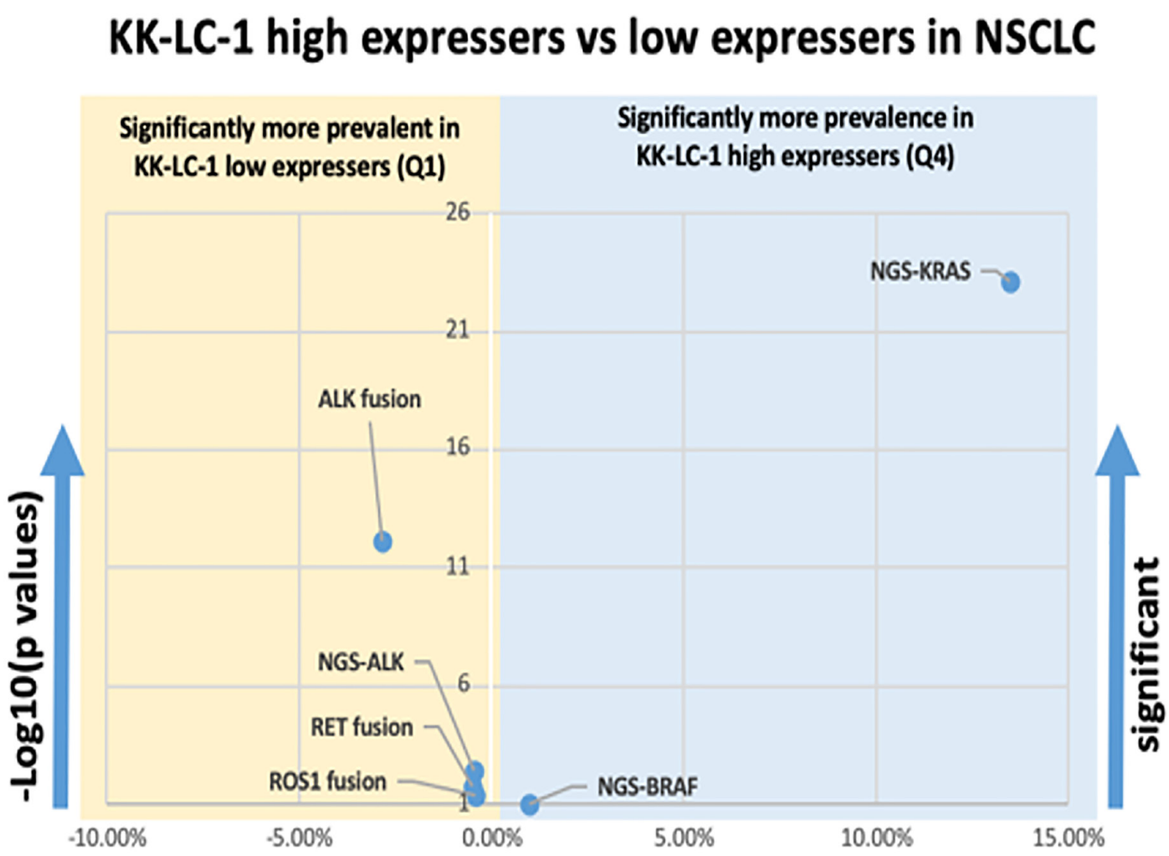

Supplementary Figure 2: KK-LC-1 high vs. low expressors in NSCLC.

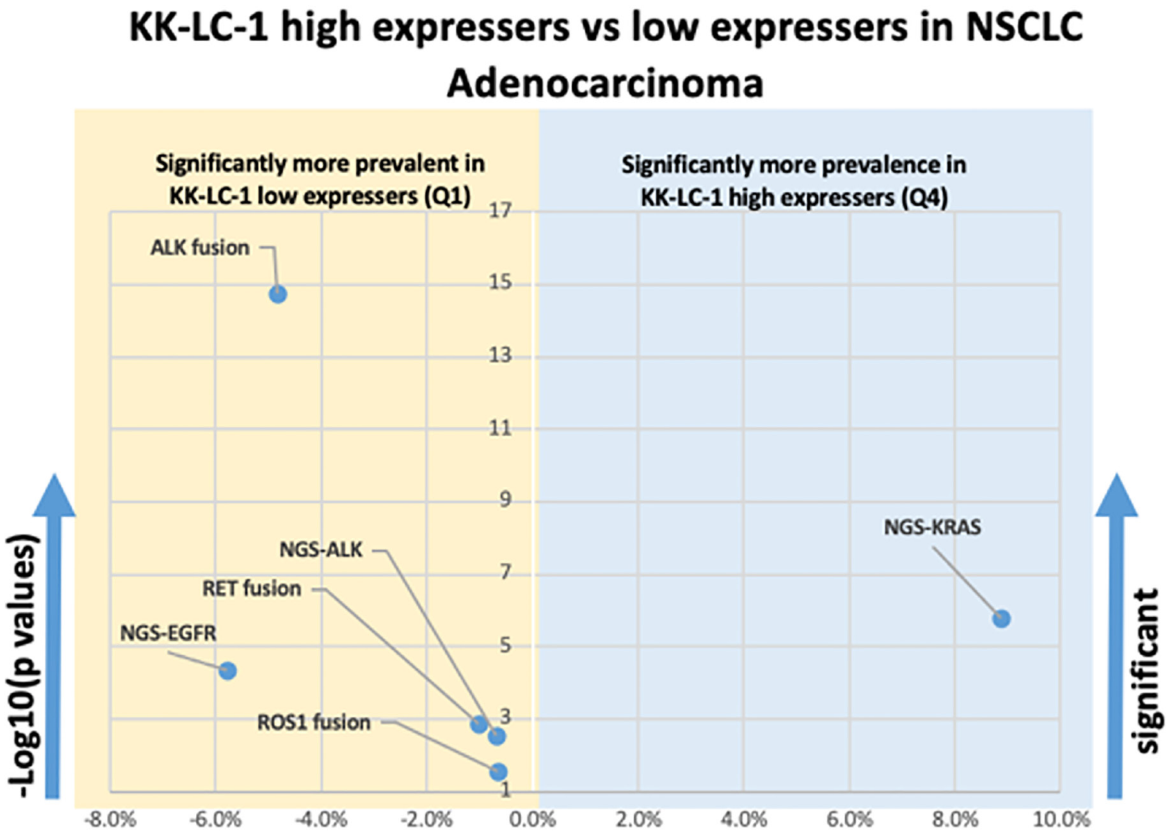

Supplementary Figure 3: KK-LC-1 high vs. low expressors in lung adenocarcinoma.

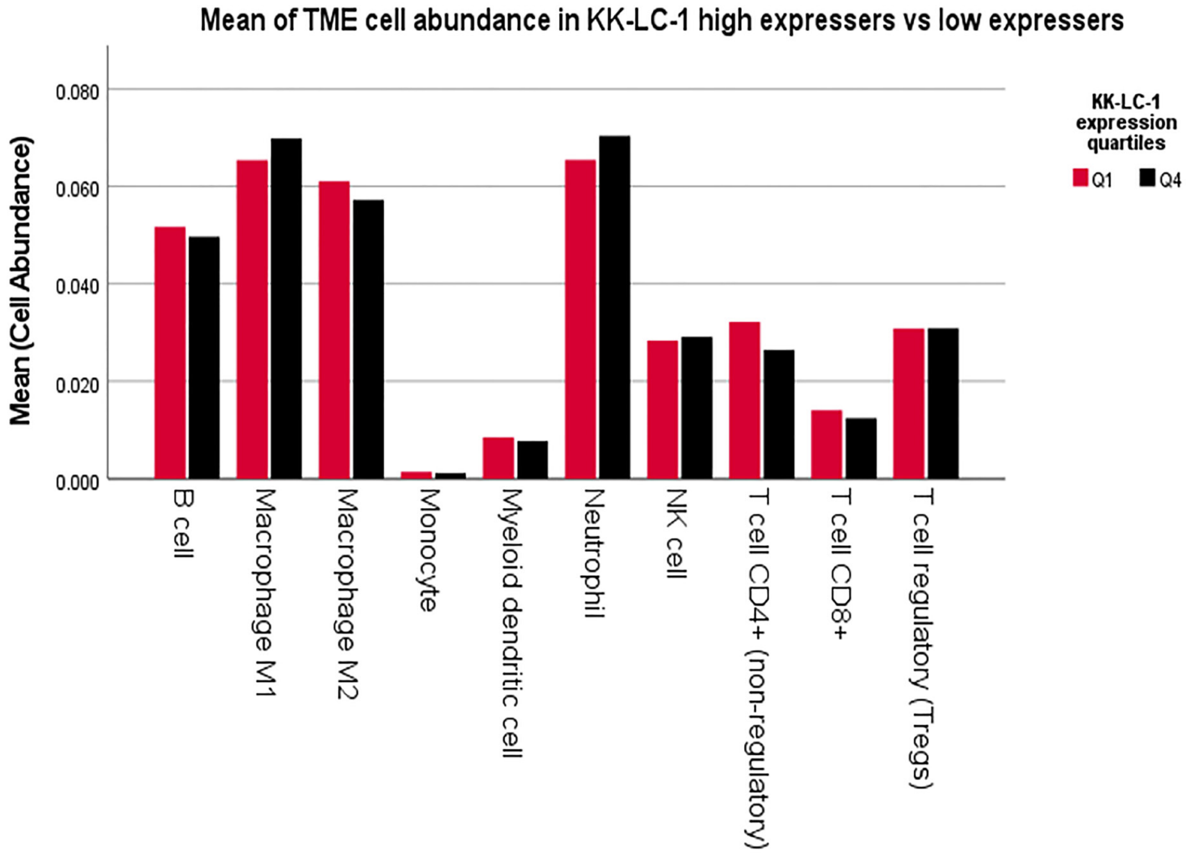

Supplementary Figure 4: Tumor microenvironment (TME) mean values in adenocarcinoma.

Supplementary Table 1: KK-LC-1 expression quartiles defined cutoffs

KK-LC-1 Quartile	TPM range
Q1	0–0.553
Q2	0.554–2.54
Q3	2.55–11.78
Q4	11.79–265.76

Supplementary Table 2: Pathology of tumors stratified by KK-LC-1 expression quartiles

KK-LC-1 expression quartiles	Adenocarcinoma *N* (%)	Adenosquamous *N* (%)	Squamous *N* (%)	unclear or mixed *N* (%)	Total *N* (%)
**Q1**	1294 (52.9)	32 (1.3)	877 (35.8)	245 (10.0)	2448 (25.0)
**Q2**	1578 (64.5)	17 (0.7)	623 (25.5)	229 (9.4)	2447 (25.0)
**Q3**	1603 (65.5)	21 (0.9)	540 (22.1)	283 (11.6)	2447 (25.0)
**Q4**	1730 (70.7)	23 (0.9)	448 (18.3)	247 (10.1)	2448 (25.0)
**Total**	**6205 (63.4)**	**93 (0.9)**	**2488 (25.4)**	**1004 (10.3)**	**9790**

Supplementary Table 3: KRAS mutation distribution among KK-LC-1 expression quartiles

KK-LC-1 Quartile	G12C (%)	G12 Other (%)	G13(Any) (%)	Other (%)	Total
Q1	178 (36.5)	230 (47.1)	30 (6.1)	50 (10.2)	488
Q2	257 (39.7)	300 (46.4)	43 (6.6)	47 (7.3)	647
Q3	321 (41.8)	336 (43.8)	60 (7.8)	51 (6.6)	768
Q4	338 (42.8)	320 (40.6)	61 (7.7)	70 (8.9)	789
**Total**	**1094**	**1186**	**194**	**218**	**2692**

Supplementary Table 4: *EGFR* mutation distribution among KK-LC-1 expression quartiles

KK-LC-1 Quartile	Sensitizing mutation (%)	Resistant mutation (%)	Both Resistant and sensitizing (%)	Other (%)	Total
Q1	245 (86.9)	35 (12.4)	0 (0)	2 (0.7)	282
Q2	313 (89.9)	34 (9.8)	0 (0)	1 (0.3)	348
Q3	240 (88.6)	28 (10.3)	0 (0)	3 (1.1)	271
Q4	221 (88.8)	27 (10.8)	1 (0.4)	0 (0)	249
**Total**	**1019**	**124**	**1**	**6**	**1150**

Supplementary Table 5: *BRAF* mutation distribution among KK-LC-1 expression quartiles

KK-LC-1 Quartile	Class 1	Class 2	Class 3	Other	Total
Q1	25 (30.9)	18 (22.2)	25 (30.9)	13 (16.0)	81
Q2	38 (45.2)	19 (22.6)	17 (20.2)	10 (11.9)	84
Q3	29 (28.7)	26 (25.7)	31 (30.7)	15 (14.9)	101
Q4	33 (32.0)	33 (32.0)	22 (21.4)	15 (14.6)	103
**Total**	**125**	**96**	**95**	**53**	**369**

Supplementary Table 6: *BRAF* fusion partners distribution among KK-LC-1 expression quartiles

KK-LC-1 quartiles	AGAP3	AGK	AHNAK	AKAP9	DOCK4	MIPOL1	MKRN1	NRF1	SND1	TAX1BP1	TRIM24	Total
Q1	1				2							3
Q2		1						1	1	1		4
Q3		1					1		1			3
Q4	1	1	1	1		1					1	6
**Total**	**2**	**3**	**1**	**1**	**2**	**1**	**1**	**1**	**2**	**1**	**1**	**16**

Supplementary Table 7: *EGFR* mutation criteria

**EGFR Sensitizing Mutations**
Any Activating mutation
G719X/S768I/L861Q
Exon 19 deletion
Exon 21 (L858R)

**EGFR Resistant Mutation**
Exon 20 insertion mutations
T790M mutation

Supplementary Table 8: *BRAF* mutation criteria

**BRAF mutation**	**BRAF Class**
V600E	Class 1
G464V	Class 2
G469A	
G469R	
G469V	
K601E	
K601N	
L597Q	
L597V	
D594G	Class 3
D594N	
F595L	
G466A	
G466E	
G466V	
G469E	
G596R	
N581I	
N581S	
S467L	
